# High-luminosity meV-resolution single-shot hard X-ray spectrograph for cavity-based X-ray free-electron lasers

**DOI:** 10.1107/S1600577525004278

**Published:** 2025-06-12

**Authors:** Keshab Kauchha, Peifan Liu, Paresh Pradhan, Yuri Shvyd’ko

**Affiliations:** a Argonne National Laboratory Lemont IL60439 USA; RIKEN SPring-8 Center, Japan

**Keywords:** X-rays, Bragg diffraction, angular dispersion, spectrograph, cavity-based XFELs

## Abstract

Cavity-based X-ray free-electron lasers (CBXFELs) represent a possible realization of fully coherent hard X-ray sources having high spectral brilliance along with a narrow spectral bandwidth of ∼1–50 meV, a high repetition pulse rate of ∼1 MHz, and good stability. A diagnostic tool is required to measure CBXFEL spectra with meV resolution and high luminosity on a shot-to-shot basis. We have designed a high-luminosity single-shot hard X-ray spectrograph that images 9.831 keV X-rays in a ∼200 meV spectral window with a spectral resolution of a few meV. The spectrograph is designed around angular dispersion of X-rays in Bragg diffraction from crystals. It operates close to design specifications, exhibiting a linear dispersion rate of ∼1.4 µm meV^−1^ and a ∼200 meV window of high-fidelity spectral imaging. The experimentally demonstrated spectral resolution is ∼20 meV; this resolution is twice as low as expected from theory primarily because the spectrograph is highly sensitive to crystal angular instabilities. The experiment was performed at the bending magnet X-ray optics testing beamline 1-BM at the Advanced Photon Source.

## Abstract 2

Theoretical background, optical design, experimental test results, and numerical simulations of the high-luminosity meV-resolution single-shot hard X-ray spectrograph for spectral diagnostics of cavity-based X-ray free-electron lasers are presented.

## 1. Introduction and principles of spectrographs

With the advent of high-gain, single-pass X-ray free-electron lasers (XFELs) (Emma *et al.*, 2010 ▸; Ishikawa *et al.*, 2012 ▸; Kang *et al.*, 2017 ▸; Decking *et al.*, 2020 ▸) exhibiting extreme brightness, transverse coherence, and ultra-short pulse length, a broad range of new scientific applications became possible, extending from investigation of femtosecond dynamics of atomic and molecular systems (Bostedt *et al.*, 2013 ▸; Callegari *et al.*, 2021 ▸) to detection of long-lived ultra-narrow nuclear resonances (Shvyd’ko *et al.*, 2023 ▸).

Initially, XFELs were based on a self-amplified spontaneous emission (Kondratenko & Saldin, 1980 ▸; Bonifacio *et al.*, 1984 ▸) (SASE) process starting from shot noise and therefore having poor longitudinal coherence of X-ray pulses. Over time, various approaches have been used to improve the longitudinal coherence. Among them have been various self-seeding schemes (Feldhaus *et al.*, 1997 ▸; Saldin *et al.*, 2001 ▸; Geloni *et al.*, 2011 ▸; Amann *et al.*, 2012 ▸; Inoue *et al.*, 2019 ▸; Nam *et al.*, 2021 ▸; Liu *et al.*, 2023 ▸). A somewhat different strategy is the cavity-based X-ray free-electron laser (CBXFEL), which improves coherence by using X-ray feedback from a narrow-band X-ray cavity, such as a low-gain X-ray FEL oscillator (XFELO) (Kim *et al.*, 2008 ▸; Kim & Shvyd’ko, 2009 ▸; Lindberg *et al.*, 2011 ▸) or a high-gain X-ray regenerative amplifier FEL (XRAFEL) (Huang & Ruth, 2006 ▸; Marcus *et al.*, 2017 ▸; Freund *et al.*, 2019 ▸; Marcus *et al.*, 2020 ▸). This strategy holds promise for producing high-brilliance X-rays not only with full coherence and a high repetition rate of ∼1 MHz but also with good stability. The CBXFEL pulses are expected to have a narrow energy bandwidth, which can be as small as a few meV for XFELOs, although with about 10^2^ times smaller pulse energy of ∼10 µJ (Kim *et al.*, 2008 ▸; Kim & Shvyd’ko, 2009 ▸; Lindberg *et al.*, 2011 ▸).

To determine the performance of a source having such a narrow bandwidth, a new diagnostic tool is required — a spectrograph capable of imaging photon spectra in a single measurement. This spectrograph will measure CBXFEL spectra with meV resolution and high luminosity on a shot-to-shot basis.

**Figure 1:**
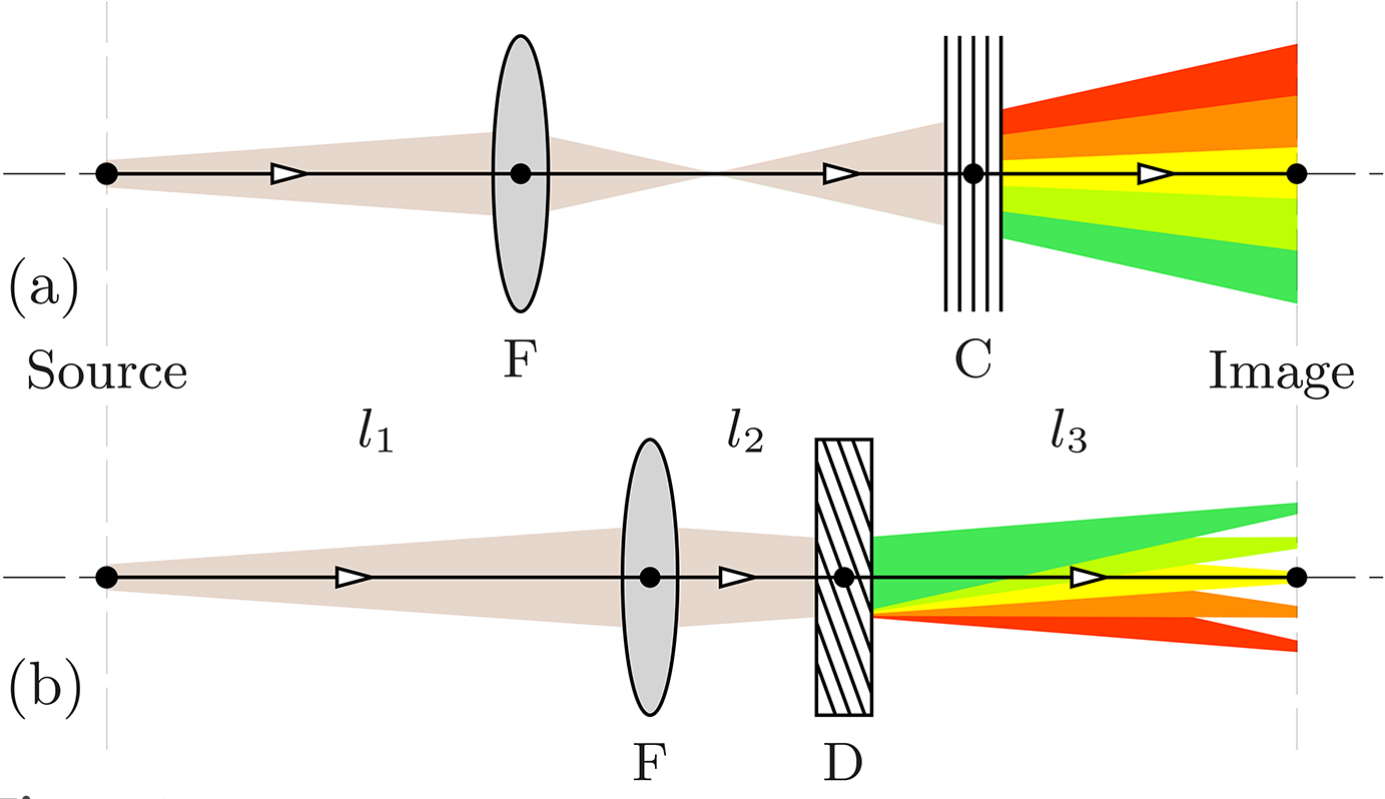
Schematics of spectrographs for imaging photon spectra from X-ray sources in a single shot. (*a*) Spectral filter spectrograph with Bragg reflecting crystal C and focusing element F. (*b*) Angular dispersive spectrograph with dispersing element D — a Bragg reflecting crystal with reflecting atomic planes at a nonzero angle to the crystal surface — and focusing element F.

Spectrographs have been designed (Yabashi *et al.*, 2006 ▸; Zhu *et al.*, 2012 ▸) and demonstrated (Yabashi *et al.*, 2006 ▸; Zhu *et al.*, 2012 ▸; Terentyev *et al.*, 2016 ▸; Boesenberg *et al.*, 2017 ▸) and are in use as hard X-ray spectral diagnostic tools (Zhu *et al.*, 2012 ▸; Kujala *et al.*, 2020 ▸) for the current generation of high-gain, single-pass XFELs (Emma *et al.*, 2010 ▸; Amann *et al.*, 2012 ▸; Ishikawa *et al.*, 2012 ▸; Inoue *et al.*, 2019 ▸; Decking *et al.*, 2020 ▸; Nam *et al.*, 2021 ▸; Liu *et al.*, 2023 ▸). These devices use X-ray Bragg diffraction from crystals; namely, they exploit the fact that, when diffraction occurs at a particular incidence angle θ (Bragg’s angle), X-rays of a specific energy are filtered out (reflected). That is, for photon incidence at Bragg’s angle θ to reflecting atomic planes with interplanar distance *d*_*H*_, exclusively X-rays of specific photon energy *E* = 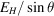 (Bragg’s law) are reflected, where *E*_*H*_ = *hc*/2*d*_*H*_. Fig. 1 ▸(*a*) shows a schematic of such a ‘spectral filter’ spectrograph. Despite a very small angular divergence (∼µrad) of the XFEL beams, these devices can have a significant spectral window of imaging of 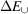 ≃ 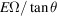 ≃ 50 eV. This large spectral window is a consequence of artificially introducing a large variation (Ω ≃ 1 mrad) in the incidence angle θ either by focusing X-rays on the crystal (Yabashi *et al.*, 2006 ▸) or by bending the crystal (Zhu *et al.*, 2012 ▸). The energy resolution of such spectrographs is limited by the Bragg reflection bandwidth Δ*E*, which is typically ∼1–0.1 eV. The corresponding small angular acceptance [Δθ = 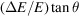 ≃ 1–10 µrad] results in low luminosity (∝ Δθ/Ω ≃ 10^−3^–10^−2^).

Spectrographs for CBXFELs must have a better spectral resolution and higher luminosity than available from current spectral filter spectrographs in use at XFELs. To this end, we apply here an alternative spectrographic approach that uses angular dispersion of X-rays in Bragg diffraction from a crystal (Shvyd’ko, 2004 ▸; Shvyd’ko *et al.*, 2006 ▸) — a hard X-ray grating effect — combined with focusing (Kohn *et al.*, 2009 ▸; Shvyd’ko, 2015 ▸).

The operating principle and basic components of the angular dispersive spectrograph are presented schematically in Fig. 1 ▸(*b*). Dispersing element D is a broadband Bragg crystal reflector in asymmetric scattering geometry with reflecting atomic planes at nonzero (asymmetry) angle η to the crystal surface. This dispersing element reflects X-rays of different photon energies *E* at different angles θ′ with the angular dispersion rate 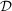 = dθ′/d*E* similar to a diffraction grating (the angular dispersion effect). Finally, focusing element F focuses different spectral components on different locations on the image plane, located at distance *l*_3_ from the dispersing element. In the general case, the dispersing element can be composed of several crystals (as in the present paper) exhibiting a cumulative dispersion rate 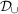 larger than that of a single reflector (Shvyd’ko *et al.*, 2013 ▸). Unlike the spectral filtering spectrographs in Fig. 1 ▸(*a*), all photons from the source are captured (within the spectral or angular acceptance ranges of the optics), thus ensuring high luminosity. In addition, the spectral resolution 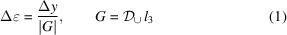
relies on the magnitude of the linear dispersion rate *G* = d*y*/d*E* in the image plane and on the tightness of the monochromatic focal spot size Δ*y* [see Shvyd’ko (2015 ▸), Appendix *A*[App appa], and equation (11)] rather than on the smallness of the Bragg reflection bandwidth.

A proof-of-principle angular dispersive hard X-ray spectrograph was demonstrated by Shvyd’ko *et al.* (2013 ▸), who also showed that multiple crystal arrangements can enhance the cumulative angular dispersion rate of the system 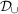, which is critical for achieving high spectral resolution [equation (1)]. Practical spectrographs were demonstrated for applications in nuclear resonance (Chumakov *et al.*, 2019 ▸) and resonant inelastic scattering (RIXS) (Bertinshaw *et al.*, 2021 ▸) spectroscopies.

The spectrograph presented in this publication was designed to characterize X-ray pulses of a CBXFEL demonstrator (Marcus *et al.*, 2019 ▸; Liu *et al.*, 2024*a* ▸; Liu *et al.*, 2024*b* ▸), which is being developed through a joint project of Argonne National Laboratory, SLAC National Accelerator Laboratory, and RIKEN. The CBXFEL will be driven by electron beams of the Linac Coherent Light Source (LCLS) facility at SLAC (Emma *et al.*, 2010 ▸) and operated at a fixed nominal photon energy of *E* = 9.831 keV to produce X-ray pulses with a bandwidth of ≲60 meV. These parameters determine the design values of the spectrograph: photon energy *E*, spectral window of imaging Δ*E*_∪_ ≳ 100 meV, and spectral resolution Δɛ ≃ 10 meV. In the following discussion, we present design details of the spectrograph and results of tests performed at X-ray optics beamline 1-BM at the Advanced Photon Source (APS) at Argonne National Laboratory (Macrander *et al.*, 2016 ▸).

## 2. Optical design and components of the spectrograph

**Figure 2:**
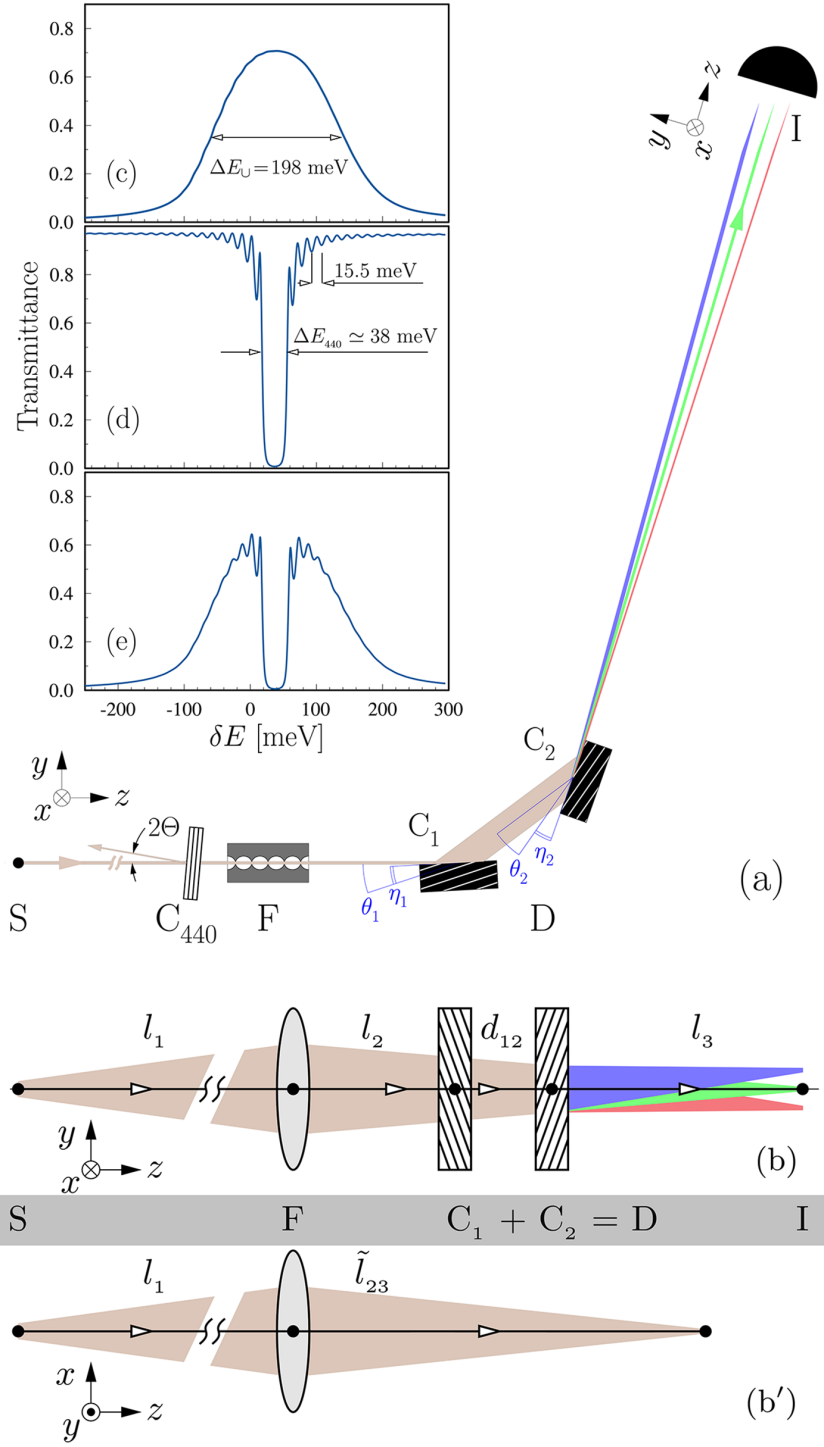
Schematics and theoretical spectral profiles of the angular dispersive spectrograph. (*a*) Optical scheme shown in the diffraction (*y*, *z*) plane with X-ray source S, focusing element F, double-crystal dispersing element D composed of crystals C_1_ and C_2_, and X-ray imager I. Crystal C_440_ is a spectral resolution probe; see panel (*d*). (*b*)–(*b*′) Equivalent unfolded optical schemes in the (*y*, *z*) diffraction (*b*) and (*x*, *z*) sagittal (*b*′) planes. (*c*)–(*e*) Theoretical spectral profiles of X-rays passing through (*c*) crystals C_1_–C_2_; (*d*) a spectral resolution probe — a narrow-band diamond Bragg back-reflecting crystal C_440_; and (*e*) through both C_440_ and C_1_–C_2_. See text for details.

To achieve the needed spectral resolution of Δɛ ≃ 10 meV, a dispersing element D with an angular dispersion rate of 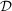 ≃ 1 µrad meV^−1^ is required [see equation (1)], assuming Δ*y* ≃ 10 µm and *l*_3_ ≃ 1 m. This performance can be realized by using a rather simple dispersing element D composed of two asymmetrically cut crystals C_1_ and C_2_ in the mirror-symmetric dispersive (++) arrangement, as shown in the optical scheme of the spectrograph in Fig. 2 ▸(*a*). The spectrograph is shown in the diffraction plane (*y*, *z*), which coincides with the angular dispersion plane.

To achieve the target value of the cumulative dispersion rate 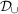, the low-index 220 Bragg reflections from germanium (Ge) crystals are used, with the parameters provided in Table 1 ▸. The use of Ge, rather than the more standard Si (silicon) crystals, makes it possible to maximize the spectral window of imaging to Δ*E*_∪_ = 198 meV and the angular acceptance.^1^ The calculated spectral profile of the X-rays reflected by the crystals^2^ is shown in Fig. 2 ▸(*c*).

Dispersing elements in the same configuration were used previously by Chumakov *et al.* (2019 ▸) and Bertinshaw *et al.* (2021 ▸). This is the simplest configuration that ensures enhancement of the cumulative rate 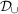 versus dispersion rate of a single reflector. Simultaneously, it leaves unchanged the beam cross-section at the exit of the dispersing element, as the cumulative asymmetry factor *b*_∪_ is 1 in this case.

The focusing element F is a compound refractive lens (CRL) (Snigirev *et al.*, 1996 ▸) composed of 2D paraboloidal beryllium lenses (Lengeler *et al.*, 1999 ▸) with parameters presented in Table 1 ▸.

The spectrograph design parameters, also shown in Table 1 ▸, are optimized using the equations of the spectrograph theory (Shvyd’ko, 2015 ▸) that are presented in a focused form in Appendix *A*[App appa]. We also show there that the nonzero distance *d*_12_ between crystals C_1_ and C_2_ of the dispersing element leads to astigmatism: the focal points of X-rays propagating in the mutually perpendicular (*y*, *z*) diffraction and (*x*, *z*) sagittal planes are spaced by ∼*d*_12_ along the optical axes, as illustrated on the equivalent unfolded schemes of the spectrograph in Figs. 2 ▸(*b*) and 2(*b*′).

To characterize the spectrograph’s performance — dispersion rates, spectral resolution, spectral window of imaging — a spectral resolution probe C_440_ is added to the setup, as shown in Fig. 2 ▸(*a*). Crystal C_440_ is an X-ray-transparent diamond crystal of thickness *d* = 40 µm in the 440 Bragg reflection close to backscattering with incidence angle Θ 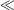 1. Inserting this crystal creates an absorption notch in the X-ray transmission spectrum having a bandwidth Δ*E*_440_ = 38 meV of the Bragg reflection. The tails of the notch display fringes of equal thickness with a periodicity of *hc*/2*d* = 15.5 meV. The calculated spectrum is shown in Fig. 2 ▸(*d*). Fig. 2 ▸(*e*) presents the result of the combined action of C_1_–C_2_ and C_440_ on the spectrum of X-rays propagating through the system. The energy variation of the absorption notch location, 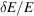 = 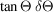, is controlled by variation of the incidence angle δΘ according to Bragg’s law; this relationship enables energy calibration of the spectrograph. The backscattering geometry ensures that the spectral profile of the notch is insensitive to the angular divergence of the X-ray beam and also permits straightforward measurement of Θ.

In the experiment, a double-crystal Si(111) monochromator is used upstream of C_440_ to reduce the bandwidth of X-rays to about 1 eV (Macrander *et al.*, 2016 ▸). This monochromator is not shown in Fig. 2 ▸(*a*). The spectral flux density of photons provided to the experiment is ∼10^9^ photons s^−1^ eV^−1^ mm^−2^.

## 3. Experiment

**Figure 3:**
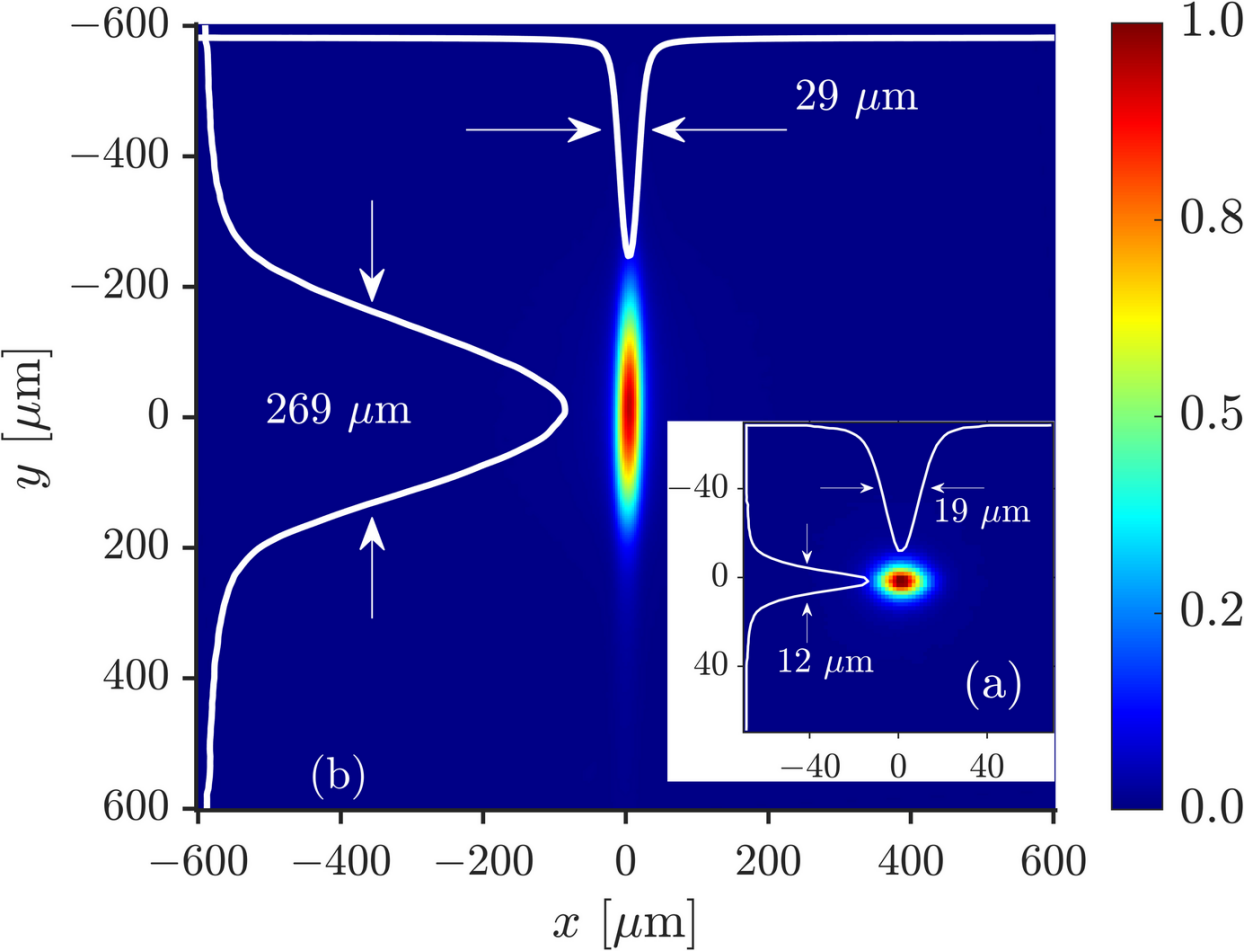
Images of the monochromated X-ray beam from a bending magnet source taken in (*a*, inset) inline focusing configuration S–F–I of Fig. 2 ▸(*b*′), and (*b*) in spectrograph configuration S–F–C_1_–C_2_–I of Figs. 2 ▸(*a*) and 2(*b*) with the double-crystal element D (C_1_–C_2_) dispersing X-rays in the diffraction plane (*y*-direction). Also shown are beam profiles *S*(*y*) and *S*(*x*), which are a result of integrating the images over *x* and *y*, respectively.

In the first measurement, the monochromatic X-ray source image size was determined to assess the energy resolution that can be achieved under the given experimental conditions of beamline 1-BM (Macrander *et al.*, 2016 ▸) at APS. Fig. 3 ▸(*a*) shows the source image with a full width at half-maximum (FWHM) of Δ*x*_e_′ × Δ*y*_e_′ = 19 µm × 12 µm. It was measured in the inline S–F–I configuration excluding the dispersing element as in the equivalent scheme of Fig. 2 ▸(*b*′), with *l*_1_ and 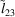 values from Table 1 ▸. Given the 1 eV bandwidth of the X-rays, focusing by the CRL can be considered achromatic, and therefore the measured sizes correspond to the monochromatic source image size. Both horizontal and vertical values, and especially the vertical [see Fig. 3 ▸(*a*)], are larger than the design monochromatic image size Δ*x*′ × Δ*y*′ given in Table 1 ▸. This broadening occurs mostly because of imperfections in the upstream beamline optical components, *e.g.* the Si(111) monochromator and Be windows, and the limited spatial resolution ∼6 µm of the YAG:Ce-based scintillator X-ray imager. The imager uses a low-noise Andor Zyla 4.2 Plus camera and Infinity KC VideoMax optic with IF-3.5 objective (Liu *et al.*, 2024*a* ▸). The vertical size determines the smallest spectral resolution that can be expected under conditions of the present experiment: Δɛ_e_ ≃ 8 meV (see Table 1 ▸).

In the next step, the dispersing element was added to complete the spectrograph. Fig. 3 ▸(*b*) demonstrates its immediate effect: the *y*-image size is greatly enlarged from 12 µm to 269 µm (FWHM). The image was taken in the location of the diffraction plane focus, which is away from the sagittal focus by about *d*_12_ = 15 cm due to astigmatism in the system. [The best focusing in the diffraction and sagittal planes is determined by lens equations (21) and (24), respectively; see also the numerical simulations discussed in the next section.] Because of this difference in focal position, the *x*-image size in Fig. 3 ▸(*b*) is larger than that in Fig. 3 ▸(*a*). The pitch angles of crystals C_1_ and C_2_ (corresponding to θ_1_ and θ_2_) were aligned by maximizing the intensity of reflected X-rays, while the yaw and roll angles were aligned to minimize the *x*-image size of the source in the sagittal image plane, which is unaffected by the angular dispersion effect. The images were made with ∼10^7^ photons (exposure time 10 s) and a signal-to-noise ratio of ∼150.

**Figure 4:**
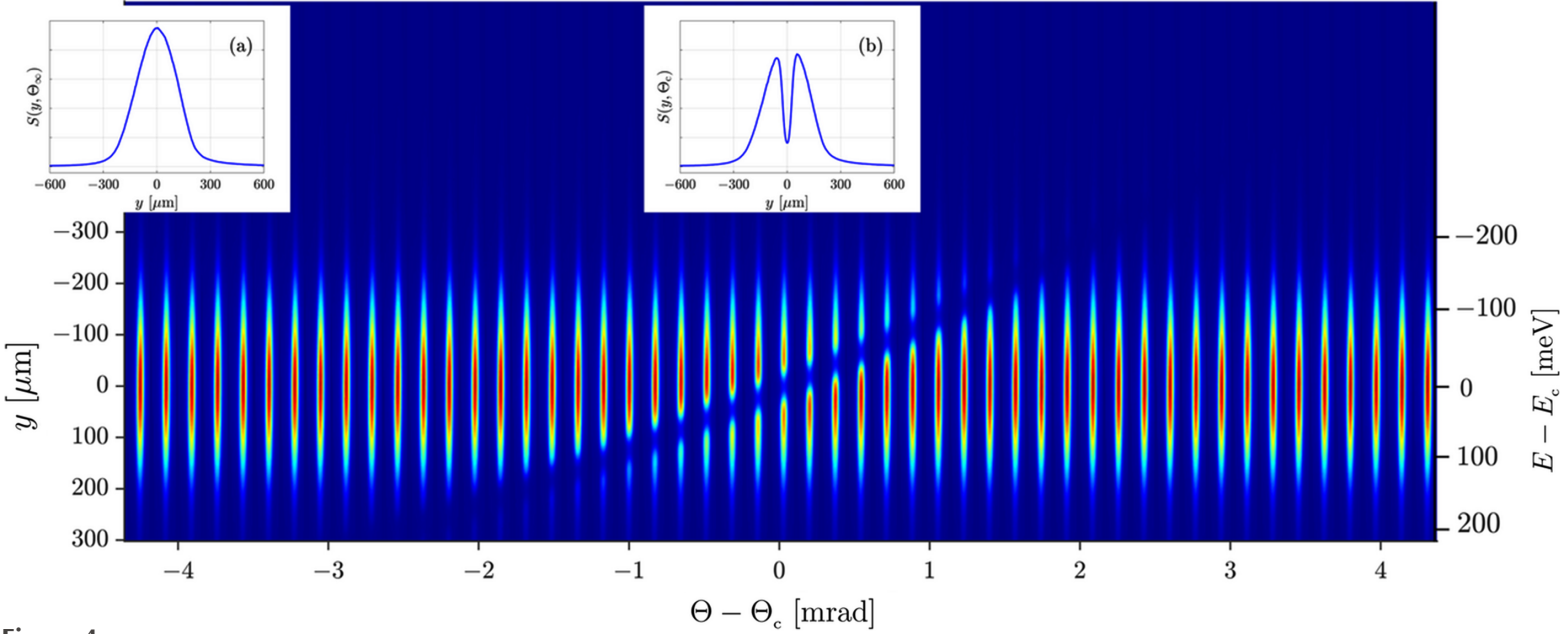
A sequence of X-ray spectrograph images similar to that in Fig. 3 ▸(*b*) but taken here with spectral resolution probe *C*_440_ in the beam and angular deviation Θ from the exact Bragg back reflection being changed incrementally. Inserts show examples of spectral profiles *S*(*y*, Θ) taken at selected angles Θ_∞_ = 4.91 mrad [inset (*a*)] and Θ_c_ = 9.26 mrad [inset (*b*)]. The Θ scale is centered at Θ_c_ = 9.259 mrad; this value corresponds to the notch in the middle of the spectral window of imaging [inset (*b*)]. The energy scale *E* − *E*_c_ = 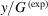 is obtained using the linear dispersion rate 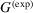 derived from these images as detailed in the discussion of Fig. 5 ▸.

Finally, the spectral resolution probe C_440_ was added to the setup to measure the dispersion rates, the spectral resolution, and the window of imaging of the spectrograph. Fig. 4 ▸ shows a sequence of X-ray spectrograph images similar to that in Fig. 3 ▸(*b*) but taken here with the spectral probe in the beam and the angular deviation Θ from exact Bragg back reflection being changed incrementally. As already noted, the effect of C_440_ is to produce an absorption notch. The spectral location of this notch (imager’s coordinate *y*) varies with Θ according to Bragg’s law. The Θ-scale is centered at Θ_c_ = 9.259 mrad; at this angle, the notch is in the middle of the spectral window of imaging of the spectrograph. Also shown are examples of spectral image profiles *S*(*y*, Θ) taken at selected angles Θ_∞_ = 4.91 mrad and Θ_c_ = 9.26 mrad [Fig. 4 ▸, insets (*a*) and (*b*), respectively].

**Figure 5:**
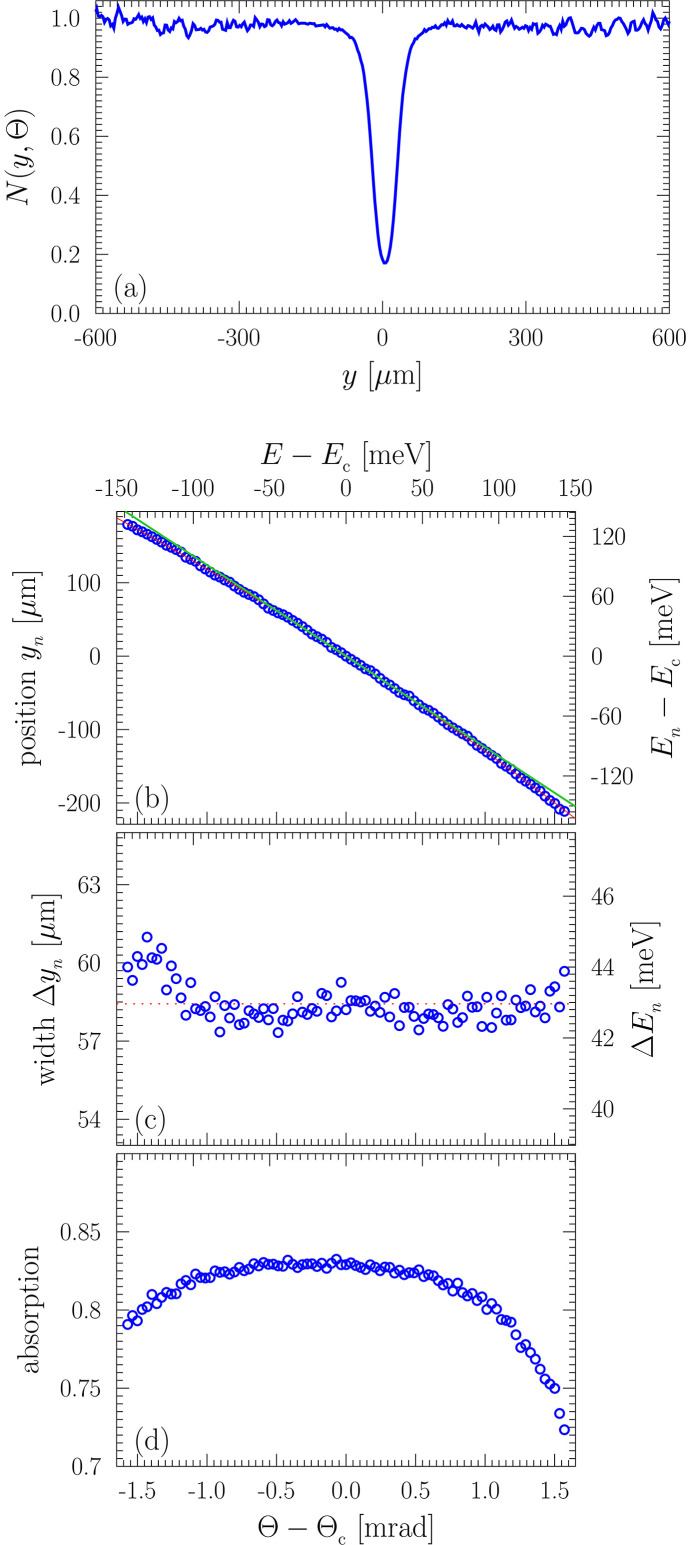
Normalized absorption notch profiles *N*(*y*, Θ) and notch parameters as a function of angular deviation Θ from exact Bragg backscattering of probe *C*_440_. (*a*) An example of the normalized absorption notch profile *N*(*y*, Θ) = *S*(*y*, Θ)/*S*(*y*, Θ_∞_): that is, the ratio of the X-ray beam spectral image profile *S*(*y*, Θ) [see Fig. 4 ▸(*b*)] to the X-ray beam image profile *S*(*y*, Θ_∞_) when unaffected by the absorption notch [see Fig. 4 ▸(*a*)]. (*b*) Absorption notch location *y*_*n*_. (*c*) Notch FWHM Δ*y*_*n*_. (*d*) Absorption effect 1 − *N*(*y*_*n*_, Θ).

Fig. 5 ▸ shows an example of a normalized absorption notch profile *N*(*y*, Θ) in panel (*a*) and plots of normalized notch parameters in panels (*b*)–(*d*) as a function of angular deviation Θ − Θ_c_ derived from the images of Fig. 4 ▸. The normalized absorption notch profile *N*(*y*, Θ) = *S*(*y*, Θ)/*S*(*y*, Θ_∞_) is defined as the ratio of the X-ray beam spectral image profile *S*(*y*, Θ) at a particular angle Θ [see Fig. 4 ▸(*b*)] to the X-ray beam image profile *S*(*y*, Θ_∞_) when unaffected by the absorption notch [see Fig. 4 ▸(*a*)]. Figs. 5 ▸(*b*), 5 ▸(*c*), and 5(*d*) present, respectively, the position *y*_*n*_ of the absorption notch minimum, the notch width Δ*y*_*n*_ (FWHM), and absorption effect 1 − *N*(*y*_*n*_, Θ), all as a function of Θ − Θ_c_, evaluated for each normalized absorption notch profile *N*(*y*, Θ).

In Figs. 5 ▸(*b*) through 5 ▸(*d*), the bottom angular scale Θ − Θ_c_ is converted into the top energy scale *E* − *E*_c_ using the Dumond tangent *D*_t_ = d*E*/dΘ|_c_ = 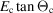 = 91 (5) meV mrad^−1^ derived from Bragg’s law at Θ = Θ_c_. The main contribution to the error of *D*_t_ is a ∼0.4 mrad inaccuracy in the determination of Θ_c_ in the experiment. The red line in Fig. 5 ▸(*b*) is a quadratic fit. The green line is its tangent*T* = d*y*_*n*_/dΘ|_c_ = −124 µm mrad^−1^ at Θ = Θ_c_. The spatial scales on the left of Figs. 5 ▸(*b*) and 5 ▸(*c*) and on Fig. 4 ▸ are converted to the energy scales on the right using the linear dispersion rate of the spectrograph, 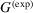 = d*y*_*n*_/d*E*|_c_ = 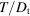 = 1.36 (7) µm meV^−1^. The value of the linear dispersion rate 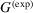 obtained from the experimental data is in good agreement with the design value *G* of Table 1 ▸.

This result allows us to determine the spectral window of imaging that corresponds to the image width Δ*y* = 269 µm in Fig. 3 ▸(*b*), namely 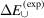 = 198 (10) meV. This value also agrees well with the design value Δ*E*_∪_ of Table 1 ▸. Importantly, this result not only presents the characteristic spectral range of the X-rays transmitted through the spectrograph but also identifies the region in which the normalized notch profile has a constant width and relatively constant absorption effect [see Figs. 5 ▸(*c*) and 5 ▸(*d*)], that is, the region where the spectral imaging has its highest fidelity.

Accordingly, the notch width measured by the imager, Δ*y*_*n*_ = 58.5 µm [Fig. 5 ▸(*c*)], can be translated to a spectral notch width of Δ*E*_*n*_ = 43 (2) meV. The latter is close to, albeit larger than, the theoretical value of Δ*E*_440_ = 38 meV of Fig. 2 ▸(*d*). This difference indicates that the spectrograph has a limited spectral resolution, which can be estimated as 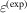 = [(Δ*E*_*n*_)^2^ − (Δ*E*_440_)^2^]^1/2^ = 20 (4) meV. This value is more than a factor of two larger than the expected value of ɛ_e_ = 8.3 meV (Table 1 ▸). It is also larger than the 15.5 meV period of the fringes seen in the theoretical spectral profiles in Figs. 2 ▸(*d*) and 2 ▸(*e*) and explains why these fringes are not observed in the experiment, *e.g.* in Fig. 5 ▸(*a*). Moreover, it is due to this limited spectral resolution that the measured absorption effect is about 83% [Fig. 5 ▸(*d*)] rather than the 99% expected from theory [Fig. 2 ▸(*d*)].

## 4. Numerical simulations and discussion

**Figure 6:**
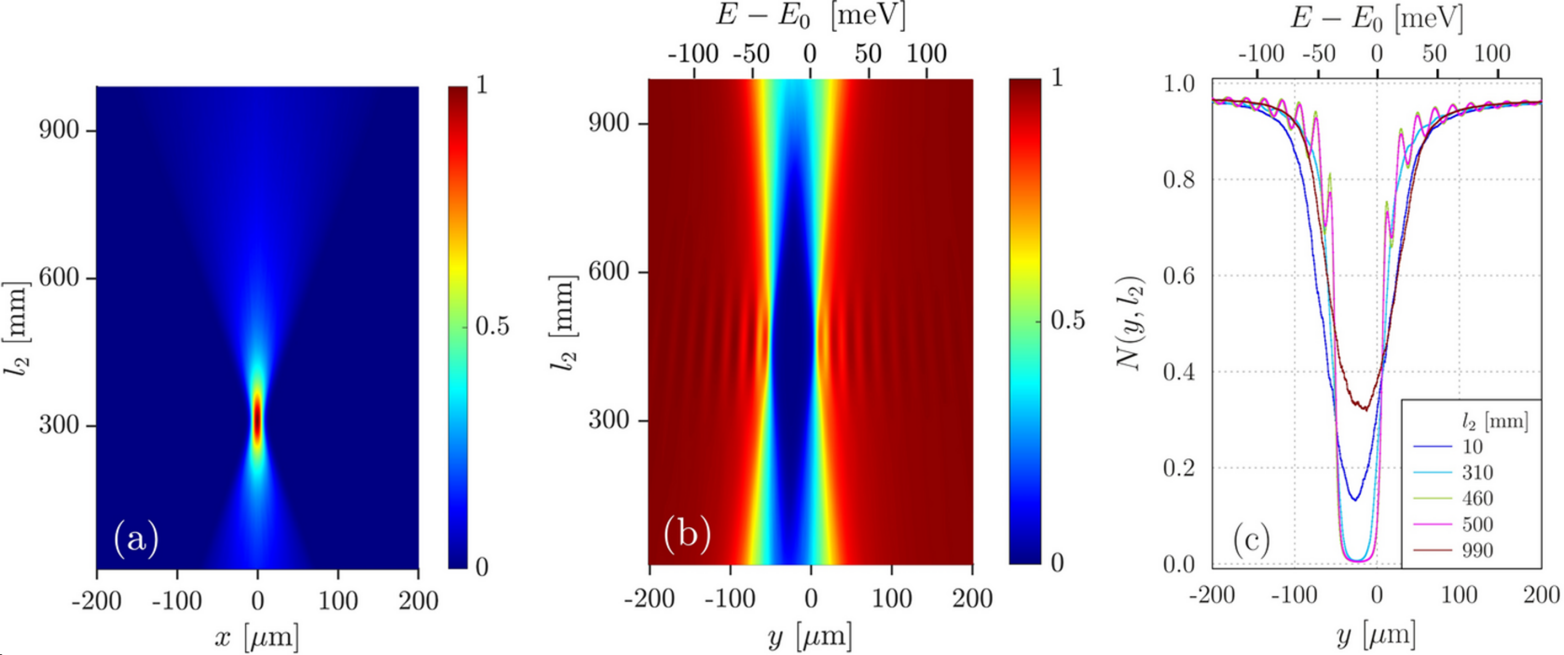
Numerical simulations of X-ray imaging by the spectrograph. (*a*) 2D color map of the image profiles *S*(*x*, *l*_2_) in the sagittal plane as a function of distance *l*_2_ between movable focusing element F (CRL) and fixed crystal C_1_. (*b*) 2D color map of the normalized absorption notch profiles *N*(*y*, *l*_2_) in the diffraction plane as a function of *l*_2_. (*c*) Examples of the normalized adsorption notch profiles *N*(*y*, *l*_2_) at particular *l*_2_.

Numerical simulation results of X-ray imaging by this angular dispersive spectrograph, shown in Fig. 6 ▸, present its ultimate performance under idealized conditions of the experiment. The simulations were carried out with the X-ray optics modeling package *Shadow3* (Sanchez del Rio *et al.*, 2011 ▸) in the *Oasys* environment (Rebuffi & Sanchez del Rio, 2016 ▸; Rebuffi & Sanchez del Rio, 2017 ▸) using the parameters given in Table 1 ▸. The locations of the X-ray source, crystals C_1_ and C_2_, and the image plane are fixed. The conditions for the best focusing in the image plane are determined from variation of distance *l*_2_ between the focusing element F (CRL) and crystal C_1_. The results of the numerical simulations show that the best focusing for rays propagating in the sagittal and diffraction planes takes place at different locations of the focusing element. The best focusing in the sagittal plane is revealed from the sharpest image size at *l*_2_ ≃ 310 mm on the 2D color map of image profiles *S*(*x*, *l*_2_) in Fig. 6 ▸(*a*). The best focusing in the diffraction plane takes place at *l*_2_ ≃ 480 mm, as reflected in the largest absorption effect and in the highest visibility of the fringes of equal thickness on the tails of the normalized absorption notch profiles *N*(*y*, *l*_2_) on the 2D color map of *N*(*y*, *l*_2_) in Fig. 6 ▸(*b*). Examples of the normalized adsorption notch profiles *N*(*y*, *l*_2_) at particular *l*_2_ are shown additionally in Fig. 6 ▸(*c*). These results are in agreement with lens equations (21) and (24) for focusing in the diffraction and sagittal planes, respectively, and in agreement with the results of the experiment.

The limited spectral resolution observed in the experiment — in particular, the inability of the spectrograph to resolve fringes of equal thickness — is likely due to the following two reasons.

First, the X-ray trajectory in the (*y*, *z*) diffraction plane is sensitive to pitch angle instabilities of crystals C_1_ and C_2_, resulting in blurring of the image profiles *S*(*y*). As we show in Appendix *B*[App appb], an angular variation ϕ of the crystal pitch angle of either of the crystals results in a shift of the spectral image by δ*y*′ ≃ *l*_3_*b*_2_ϕ, an effect aggravated by the large absolute value of |*b*_2_|. For example, an angular error of ϕ = 1 µrad of one of the crystals, which could be caused by vibrations, for example, results in a spatial shift of δ*y*′ ≃ 22 µm or in an equivalent energy shift of δ*y*′/*G* ≃ 16 meV. Such variations can blur sharp spectral features and degrade the spectral resolution. Vibrations were minimized during the experiment but were not eliminated completely.

Second, imperfect roll and yaw angular alignment of crystals C_1_ and C_2_ results in mutually nonparallel diffraction and dispersion planes, which also degrades the resolution. Minimizing the image size in the sagittal plane by varying the roll and yaw angles, which was used in the experiment, is a tedious and equivocal procedure. It could be improved in future experiments by better pre-alignment of the crystals.

We note that crystal imperfections are unlikely to be the cause of the degradation in resolution compared with theory, since the X-ray rocking curve imaging topography (Lübbert *et al.*, 2000 ▸; Stoupin *et al.*, 2016 ▸; Pradhan *et al.*, 2020 ▸) of crystals C_1_ and C_2_ revealed high crystal quality: theoretical values for the 440 Bragg reflection width Δθ = 20.6 (6) µrad and small Bragg plane slope variations of only δθ ≃ 0.7 µrad (r.m.s.) over large crystal areas of 10 mm × 10 mm.

## 5. Summary

A high-luminosity meV-resolution single-shot hard X-ray spectrograph was designed as a CBXFEL spectral diagnostic tool to image 9.831 keV X-rays in a ∼200 meV spectral window with a spectral resolution of a few meV. The operational principle of the spectrograph is angular dispersion in Bragg diffraction from crystals. The spectrograph operates close to the design specification, exhibiting a linear dispersion rate of 1.36 µm meV^−1^ and a 200 meV window of high-fidelity spectral imaging. The experimentally obtained spectral resolution of ∼20 meV is limited by high sensitivity to crystal angular instabilities at the optics testing bending-magnet beamline 1-BM at the Advanced Photon Source, where it was tested.

## Figures and Tables

**Figure 7:**
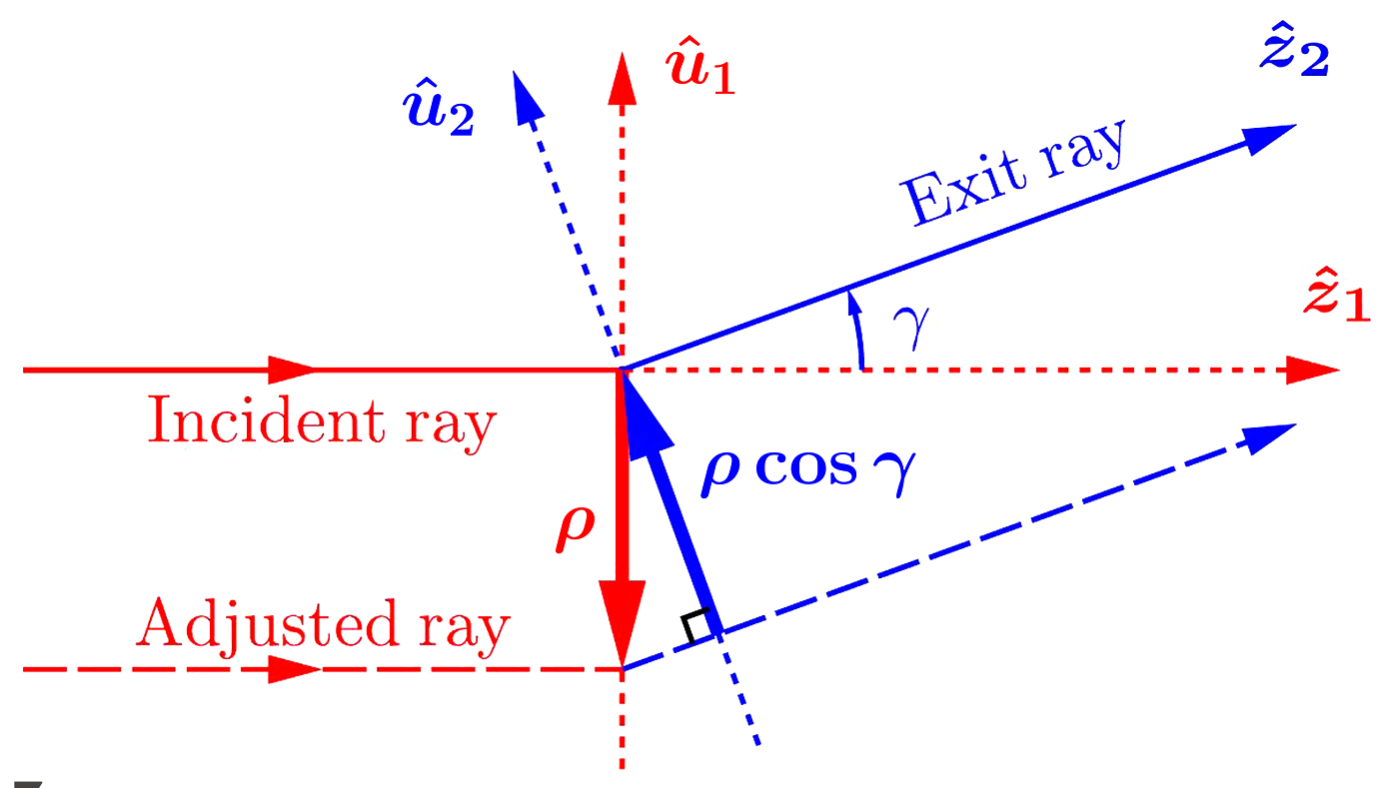
Schematic of incident and adjusted rays clarifying calculation of the X-ray trajectory distorted by a misaligned optical element (Qi & Shvyd’ko, 2022 ▸).

**Table 1:** Design parameters of X-ray sources (1-BM at APS or LCLS at SLAC); dispersing element D; focusing element F; and spectrograph Numbers in the brackets refer to the relevant equations in the text.

		Value	
	Notation or expression	1BM	LCLS
X-ray source			
Size (FWHM) (µm)	Δ*x* / Δ*y*	198 / 78	60 / 60
Photon energy (keV)	*E*	9.831 keV	

Dispersing element D and crystals C_1_, C_2_			
Bragg angles	θ_1_ = θ_2_	18.38°	
Asymmetry angles	η_1_ = −η_2_	−16.4°	
Angular acceptance (µrad)	Δθ_1_, Δθ_2_	196, 12	
Asymmetry factors	*b*_1_, *b*_2_ (2)	−0.061, −16.5	
Cumulative factor	*b*_∪_ = *b*_1_*b*_2_ (15)	1	
Angular dispersion rates (µrad meV^−1^)	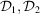 (2)	−0.032, +0.52	
Cumulative dispersion rate (µrad meV^−1^)	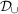 = 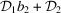 (15)	1.05	
Spectral window of imaging (meV)	Δ*E*_∪_	198	

Focusing element F (CRL) and lenses			
Be-lens radius (µm)	*R*	200	
Number of lenses	*N*_L_	16	
Focal length (m)	*f* = *R*/(2*N*_L_δ†) (Lengeler *et al.*, 1999 ▸)	1.772	

Spectrograph			
Distances between elements (m)	*l*_1_	∼35.1	∼250
	*l*_3_, *d*_12_	1.39, 0.15	
	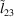 = 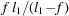 (7)	1.87	1.78
	*l*_2_ = 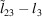 (20)	0.48	0.39
De-magnification	*A* = 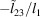 (18)	0.053	0.007
Mono. image size (µm)	Δ*x*′/Δ*y*′ (4)	10.5/4.1	0.4/0.4
Linear dispersion rate (µm meV^−1^)	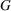 = 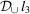 (19)	1.46	
Spectral resolution			
Ultimate (meV)	Δɛ_u_ = Δ*y*′/|*G*|. (11)	2.8	0.3
Expected (meV)	Δɛ_e_ ≃ Δ*y*_e_′/|*G*| (13)	8.3‡	

† Refractive index *n* = 1 − δ, where decrement δ = 3.52 × 10^−6^ in Be.

‡ Here, the actual monochromatic focal spot size 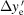 = 12 µm measured in the experiment is used to calculate the expected resolution; see Fig. 3 ▸(*a*).

## Footnotes

1 Crystals C_1_ and C_2_ were manufactured at the APS from high-quality Ge single-crystal material obtained from Umicore. The crystals’ length is 58 mm, the cross section is 22 mm × 25 mm (H × V). The beam footprint length for a 1 mm beam height – typical for our experiment – is 28 mm. See the end of Section 4 for more information on Ge crystal quality.

2 The calculations are performed for X-rays with a 100 µrad divergence introduced by focusing lens F, which is smaller that the angular acceptance of the dispersing element.

## Appendix A Spectrograph theory with an account of spacing between the dispersive crystals

A theory of the X-ray angular dispersive spectrograph was introduced by Shvyd’ko (2015 ▸). The ray-transfer matrix technique was used to derive the spectral resolution and other characteristics of the spectrograph for diverse configurations of the crystal dispersing elements and the focusing and collimating optics. The same technique is applied here to the particular case of the spectrograph in the ‘focusing-monochromator-I’ (FM1) configuration considered in the present paper, with one focusing element and the two-crystal dispersing element, as shown in the equivalent optical scheme in Fig. 2 ▸(*b*). The nonzero distance between the crystals is accurately taken into account, resulting in astigmatism in focusing.

### A1. General equations

The spectrograph in the FM1 configuration consists of the focusing element F with a focal length *f* at a distance *l*_1_ from the X-ray source S and a dispersing element with *N* crystals C_*n*_ (*n* = 1, 2,…, *N*) in asymmetric Bragg diffraction, each characterized by the asymmetry factor *b*_*n*_ and angular dispersion rate  = dθ_*n*_′/d*E*,

Here, θ and θ′ are the glancing angle of incidence (Bragg’s angle) and reflection, respectively, to the diffracting atomic planes; η is the asymmetry angle — the angle between the diffracting atomic planes and the entrance crystal surface;*E* is theX-ray photon energy. Fig. 2 ▸(*a*) shows an example of a spectrograph with a two-crystal dispersing element, while Figs. 2 ▸(*b*) and 2 ▸(*b*′) show equivalent unfolded schemes.

In the following, cumulative values are also used for the asymmetry parameter  and the angular dispersion rate ; these values result from successive Bragg reflection from *n* crystals (1 ≤ *n* ≤ *N*), which are defined as

Here *s* = −1 for clockwise and *s* = +1 for counterclockwise ray deflection upon Bragg reflection.

The first crystal C_1_ is at a distance *l*_2_ from the focusing element F, and the last crystal C_*N*_ is at a distance *l*_3_ from the image plane with position-sensitive imager I. There are nonzero distances *d*_*n*−1 *n*_ between crystals C_*n*−1_ and C_*n*_ of the dispersing element.

Following Shvyd’ko (2015 ▸), X-rays of each monochromatic spectral component *E* emerging from the source S with a lateral size Δ*x* × Δ*y* are focused to Δ*x*′ in the sagittal (*x*, *z*) plane and to Δ*y*′ in the diffraction (*y*, *z*) plane,

where the relationship between *f*, *l*_1_, *l*_2_, *d*_*n*−1 *n*_, and *l*_3_ is determined by the lens equation for the condition of best focusing,

With this equation the magnification factor in equation (4) can be presented as

Importantly, according to equation (7) the virtual lens-to-image-plane distance  is solely determined by *l*_1_, and *f* is independent of the details of the design of the dispersing element.

We assume that Bragg diffraction takes place only in the diffraction (*y*, *z*) plane. However, when considering focusing of X-rays in the sagittal (*x*, *z*) plane, which are unaffected by diffraction in the plane perpendicular to it, we have to use in this case *b*_*n*_ = −1 and  = 0 for all crystals. As a result, although lens equation (7) and the virtual distance  for the best focusing are the same in both planes, assuming the same *l*_1_ and *f*, the magnification factor [equation (8)] and the image plane locations for X-rays propagating in the diffraction (*y*, *z*) and the sagittal (*x*, *z*) planes [determined by *l*_2_, *d*_*n*−1 *n*_, *l*_3_ from equations (5) and (6)] can differ. Therefore, generally speaking, focusing systems that contain dispersing elements exhibit astigmatism: diffraction and sagittal rays form foci at different distances along the optic axis.

A change δ*E* in the X-ray photon energy *E* results in a change of the focal spot location in the diffraction (*y*, *z*) plane by

as a result of the angular dispersion of X-rays in Bragg diffraction from the asymmetrically cut crystals. Here, *G* is the linear dispersion rate of the spectrograph.

An energy variation, which results in a change |δ*y*| of the location of the source image [equation (9)] that is equal to the monochromatic source image size Δ*y*′ [equations (4)–(7)] determines the energy resolution of the spectrograph,

Using equation (8) for the magnification factor, the expression for the ultimate spectral resolution of the spectrograph (in the FM1 configuration) can be presented via the source *y*-size as follows,

The monochromatic source image size  in an actual experiment can be larger than the ultimate value Δ*y*′ given by equation (8) because of imperfections in the optical elements of the spectrograph or limited imager spatial resolution. In this case, the energy resolution of the spectrograph is given by

### A2. Two-crystal mirror-symmetric arrangement

In a particular case of the two-crystal (*N* = 2) dispersing element in mirror-symmetric dispersive (++) arrangement schematically presented in Fig. 2 ▸, the crystal angles are

In this case, using equation (3), the cumulative values are given by

Further, the expression for the virtual lens-to-image plane distance,

is obtained from equations (5) and (6) and  = . Furthermore, the linear dispersion rate

is obtained from equations (9) and (10) and  = , while the expression for the magnification factor

is derived from equations (8) and (15).

If the crystal asymmetry is large, *i.e.* −*b*_1_ = −1/*b*_2_ 1, and the distance between the crystals is small, *i.e.* (as in our experiment), then the term  can be omitted in equation (16). The linear dispersion term  in equation (17) can also be neglected resulting in a simplified expression,

In this case, *d*_12_ does not enter also the virtual lens-to-image plane distance

and it appears as if distance *d*_12_ has no impact either on the lens equation (7) or on the ultimate spectral resolution equation (12), which now read as

and

respectively.

On the other hand, in the symmetric case when *b*_1_ = *b*_2_ = −1, the virtual lens-to-image plane distance becomes

*i.e.* is exactly equal to the length of the optical path from the lens to the imager, with the lens equation taking the expected form

Here we emphasize that the virtual distance  is an invariant. What changes is the relationship between *l*_2_ and *l*_3_ and thus the location of the image planes. In relation to our experiment, these results mean that there are two different image planes for focusing of the rays in the diffraction plane (*y*, *z*) and in the sagittal (*x*, *z*) plane perpendicular to it. The sagittal image plane location is defined by focusing equation (24), while the diffraction image plane location is defined by focusing equation (21) and is *d*_12_ further away from the second crystal than the sagittal image plane, as shown in the equivalent unfolded schemes in Figs. 2 ▸(*b*) and 2 ▸(*b*′).

## Appendix B Ray trajectory distortions caused by angular errors of C1 and C2 crystals

In this section we study how much an angular misalignment ϕ of either crystal C_1_ or C_2_ of the dispersing element of the spectrograph can change the X-ray trajectory and therefore the image location in the imager plane. In practical terms, this knowledge determines tolerances on angular stability of the dispersing element crystals.

### b1. General equation

The ray-transfer matrix approach can be used to propagate rays through optical systems with misaligned optical elements (Siegman, 1986 ▸; Qi & Shvyd’ko, 2022 ▸).

Fig. 7 ▸ [adapted from Qi & Shvyd’ko (2022 ▸)] illustrates the equations used to calculate misalignments. Suppose an optical element (a crystal or a lens) is displaced from the perfect configuration by a distance ρ and an angle ϕ. The distorted propagation of the X-rays can be calculated by (i) displacing the incident X-ray presented by vector **r**_1_ with spatial and angular coordinates *u*_1_ and *v*_1_, respectively, by the same amount as the optical element but in the opposite direction (−ρ and −ϕ); (ii) transferring the ray with an appropriate matrix  of the optical element; and (iii) moving the transferred ray back by the adjusted amount. As a result, the distorted ray presented by vector  with coordinates *u*_2_ and *v*_2_ are given by (Qi & Shvyd’ko, 2022 ▸)

where γ is the angle between the exit ray and the incident ray at the location of the optical element, as shown in Fig. 7 ▸. For a crystal, the exit ray angle γ = 2θ after crystal reflection with Bragg’s angle θ. For a lens, γ = 0. Note that the first adjustment of the ray position −ρ is relative to the incident ray. To restore the exit ray to its correct position, it needs to be moved by the amount .

### b2. Application to the spectrograph case with two-crystal dispersing element

We consider a simplified case, in which only a nonzero angular error ϕ is assumed, while the spatial error ρ = 0. Besides, we will trace the trajectory of only the principle ray  = 0, which is initially on the optical axis. In this case equation (25) simplifies to

We recall that the ray-transfer matrix of an asymmetrically cut crystal is (Matsushita & Kaminaga, 1980 ▸; Shvyd’ko, 2015 ▸)

where *b* is the asymmetry parameter.

In particular, if crystal C_2_ is misaligned with an angular error ϕ, the Bragg reflected ray is

This means that the reflected ray propagates at a deflection angle Ψ_2_ = (1 − *b*_2_)ϕ to the optical axis. In the symmetric case, for which *b*_2_ = −1, the deflection angle is Ψ_2_ = 2ϕ. However, if −*b*_2_ ≫ 1 (as in our experiment) the asymmetrically cut crystal amplifies the deflection angle by a factor of 1 − *b*_2_ ≫ 1. At a distance *l*_3_, the spatial shift from the optical axis is *l*_3_Ψ_2_ =*l*_3_(1 − *b*_2_)ϕ.

If crystal C_1_ is misaligned with an angular error ϕ, the ray after two Bragg reflections (neglecting the spacing *d*_12_) is

Although −*b*_1_ 1, and therefore the angular deflection after the first crystal (1 − *b*_1_)ϕ is relatively small, the second crystal still amplifies this deflection to Ψ_1_ = *b*_2_(1 − *b*_1_)ϕ. If −*b*_2_ ≫ 1, the magnitude of Ψ_1_ is close to Ψ_2_, but has the opposite sign. Similarly, the spatial shift from the optical axis at a distance *l*_3_ is *l*_3_Ψ_1_ = *l*_3_(1 − *b*_1_)*b*_2_ϕ.

The analytical calculations provided here are confirmed by numerical simulations with the X-ray optics modeling package *Shadow3* (Sanchez del Rio *et al.*, 2011 ▸).

## References

[bb1] Amann, J., Berg, W., Blank, V., Decker, F.-J., Ding, Y., Emma, P., Feng, Y., Frisch, J., Fritz, D., Hastings, J., Huang, Z., Krzywinski, J., Lindberg, R., Loos, H., Lutman, A., Nuhn, H.-D., Ratner, D., Rzepiela, J., Shu, D., Shvyd’ko, Y., Spampinati, S., Stoupin, S., Terentyev, S., Trakhtenberg, E., Walz, D., Welch, J., Wu, J., Zholents, A. & Zhu, D. (2012). *Nat. Photon.* **6**, 693–698.

[bb2] Bertinshaw, J., Mayer, S., Dill, F.-U., Suzuki, H., Leupold, O., Jafari, A., Sergueev, I., Spiwek, M., Said, A., Kasman, E., Huang, X., Keimer, B. & Gretarsson, H. (2021). *J. Synchrotron Rad.* **28**, 1184–1192. 10.1107/S1600577521003805 PMC8284409 34212883

[bb3] Boesenberg, U., Samoylova, L., Roth, T., Zhu, D., Terentyev, S., Vannoni, M., Feng, Y., van Driel, T. B., Song, S., Blank, V., Sinn, H., Robert, A. & Madsen, A. (2017). *Opt. Express* **25**, 2852–2862. 10.1364/OE.25.002852 29519002

[bb4] Bonifacio, R., Pellegrini, C. & Narducci, L. (1984). *Opt. Commun.* **50**, 373–378.

[bb5] Bostedt, C., Bozek, J. D., Bucksbaum, P. H., Coffee, R. N., Hastings, J. B., Huang, Z., Lee, R. W., Schorb, S., Corlett, J. N., Denes, P., Emma, P., Falcone, R. W., Schoenlein, R. W., Doumy, G., Kanter, E. P., Kraessig, B., Southworth, S., Young, L., Fang, L., Hoener, M., Berrah, N., Roedig, C. & DiMauro, L. F. (2013). *J. Phys. B At. Mol. Opt. Phys.* **46**, 164003.

[bb6] Callegari, C., Grum-Grzhimailo, A. N., Ishikawa, K. L., Prince, K. C., Sansone, G. & Ueda, K. (2021). *Phys. Rep.* **904**, 1–59.

[bb7] Chumakov, A. I., Shvyd’ko, Y., Sergueev, I., Bessas, D. & Rüffer, R. (2019). *Phys. Rev. Lett.* **123**, 097402. 10.1103/PhysRevLett.123.097402 31524474

[bb8] Decking, W., Abeghyan, S., Abramian, P., Abramsky, A., Aguirre, A., Albrecht, C., Alou, P., Altarelli, M., Altmann, P., Amyan, K., Anashin, V., Apostolov, E., Appel, K., Auguste, D., Ayvazyan, V., Baark, S., Babies, F., Baboi, N., Bak, P., Balandin, V., Baldinger, R., Baranasic, B., Barbanotti, S., Belikov, O., Belokurov, V., Belova, L., Belyakov, V., Berry, S., Bertucci, M., Beutner, B., Block, A., Blöcher, M., Böckmann, T., Bohm, C., Böhnert, M., Bondar, V., Bondarchuk, E., Bonezzi, M., Borowiec, P., Bösch, C., Bösenberg, U., Bosotti, A., Böspflug, R., Bousonville, M., Boyd, E., Bozhko, Y., Brand, A., Branlard, J., Briechle, S., Brinker, F., Brinker, S., Brinkmann, R., Brockhauser, S., Brovko, O., Brück, H., Brüdgam, A., Butkowski, L., Büttner, T., Calero, J., Castro-Carballo, E., Cattalanotto, G., Charrier, J., Chen, J., Cherepenko, A., Cheskidov, V., Chiodini, M., Chong, A., Choroba, S., Chorowski, M., Churanov, D., Cichalewski, W., Clausen, M., Clement, W., Cloué, C., Cobos, J. A., Coppola, N., Cunis, S., Czuba, K., Czwalinna, M., D’Almagne, B., Dammann, J., Danared, H., de Zubiaurre Wagner, A., Delfs, A., Delfs, T., Dietrich, F., Dietrich, T., Dohlus, M., Dommach, M., Donat, A., Dong, X., Doynikov, N., Dressel, M., Duda, M., Duda, P., Eckoldt, H., Ehsan, W., Eidam, J., Eints, F., Engling, C., Englisch, U., Ermakov, A., Escherich, K., Eschke, J., Saldin, E., Faesing, M., Fallou, A., Felber, M., Fenner, M., Fernandes, B., Fernández, J. M., Feuker, S., Filippakopoulos, K., Floettmann, K., Fogel, V., Fontaine, M., Francés, A., Martin, I. F., Freund, W., Freyermuth, T., Friedland, M., Fröhlich, L., Fusetti, M., Fydrych, J., Gallas, A., García, O., Garcia-Tabares, L., Geloni, G., Gerasimova, N., Gerth, C., Geßler, P., Gharibyan, V., Gloor, M., Głowinkowski, J., Goessel, A., Gołębiewski, Z., Golubeva, N., Grabowski, W., Graeff, W., Grebentsov, A., Grecki, M., Grevsmuehl, T., Gross, M., Grosse-Wortmann, U., Grünert, J., Grunewald, S., Grzegory, P., Feng, G., Guler, H., Gusev, G., Gutierrez, J. L., Hagge, L., Hamberg, M., Hanneken, R., Harms, E., Hartl, I., Hauberg, A., Hauf, S., Hauschildt, J., Hauser, J., Havlicek, J., Hedqvist, A., Heidbrook, N., Hellberg, F., Henning, D., Hensler, O., Hermann, T., Hidvégi, A., Hierholzer, M., Hintz, H., Hoffmann, F., Hoffmann, M., Hoffmann, M., Holler, Y., Hüning, M., Ignatenko, A., Ilchen, M., Iluk, A., Iversen, J., Iversen, J., Izquierdo, M., Jachmann, L., Jardon, N., Jastrow, U., Jensch, K., Jensen, J., Jeżabek, M., Jidda, M., Jin, H., Johansson, N., Jonas, R., Kaabi, W., Kaefer, D., Kammering, R., Kapitza, H., Karabekyan, S., Karstensen, S., Kasprzak, K., Katalev, V., Keese, D., Keil, B., Kholopov, M., Killenberger, M., Kitaev, B., Klimchenko, Y., Klos, R., Knebel, L., Koch, A., Koepke, M., Köhler, S., Köhler, W., Kohlstrunk, N., Konopkova, Z., Konstantinov, A., Kook, W., Koprek, W., Körfer, M., Korth, O., Kosarev, A., Kosiński, K., Kostin, D., Kot, Y., Kotarba, A., Kozak, T., Kozak, V., Kramert, R., Krasilnikov, M., Krasnov, A., Krause, B., Kravchuk, L., Krebs, O., Kretschmer, R., Kreutzkamp, J., Kröplin, O., Krzysik, K., Kube, G., Kuehn, H., Kujala, N., Kulikov, V., Kuzminych, V., La Civita, D., Lacroix, M., Lamb, T., Lancetov, A., Larsson, M., Le Pinvidic, D., Lederer, S., Lensch, T., Lenz, D., Leuschner, A., Levenhagen, F., Li, Y., Liebing, J., Lilje, L., Limberg, T., Lipka, D., List, B., Liu, J., Liu, S., Lorbeer, B., Lorkiewicz, J., Lu, H. H., Ludwig, F., Machau, K., Maciocha, W., Madec, C., Magueur, C., Maiano, C., Maksimova, I., Malcher, K., Maltezopoulos, T., Mamoshkina, E., Manschwetus, B., Marcellini, F., Marinkovic, G., Martinez, T., Martirosyan, H., Maschmann, W., Maslov, M., Matheisen, A., Mavric, U., Meißner, J., Meissner, K., Messerschmidt, M., Meyners, N., Michalski, G., Michelato, P., Mildner, N., Moe, M., Moglia, F., Mohr, C., Mohr, S., Möller, W., Mommerz, M., Monaco, L., Montiel, C., Moretti, M., Morozov, I., Morozov, P., Mross, D., Mueller, J., Müller, C., Müller, J., Müller, K., Munilla, J., Münnich, A., Muratov, V., Napoly, O., Näser, B., Nefedov, N., Neumann, R., Neumann, R., Ngada, N., Noelle, D., Obier, F., Okunev, I., Oliver, J. A., Omet, M., Oppelt, A., Ottmar, A., Oublaid, M., Pagani, C., Paparella, R., Paramonov, V., Peitzmann, C., Penning, J., Perus, A., Peters, F., Petersen, B., Petrov, A., Petrov, I., Pfeiffer, S., Pflüger, J., Philipp, S., Pienaud, Y., Pierini, P., Pivovarov, S., Planas, M., Pławski, E., Pohl, M., Polinski, J., Popov, V., Prat, S., Prenting, J., Priebe, G., Pryschelski, H., Przygoda, K., Pyata, E., Racky, B., Rathjen, A., Ratuschni, W., Regnaud-Campderros, S., Rehlich, K., Reschke, D., Robson, C., Roever, J., Roggli, M., Rothenburg, J., Rusiński, E., Rybaniec, R., Sahling, H., Salmani, M., Samoylova, L., Sanzone, D., Saretzki, F., Sawlanski, O., Schaffran, J., Schlarb, H., Schlösser, M., Schlott, V., Schmidt, C., Schmidt-Foehre, F., Schmitz, M., Schmökel, M., Schnautz, T., Schneidmiller, E., Scholz, M., Schöneburg, B., Schultze, J., Schulz, C., Schwarz, A., Sekutowicz, J., Sellmann, D., Semenov, E., Serkez, S., Sertore, D., Shehzad, N., Shemarykin, P., Shi, L., Sienkiewicz, M., Sikora, D., Sikorski, M., Silenzi, A., Simon, C., Singer, W., Singer, X., Sinn, H., Sinram, K., Skvorodnev, N., Smirnow, P., Sommer, T., Sorokin, A., Stadler, M., Steckel, M., Steffen, B., Steinhau-Kühl, N., Stephan, F., Stodulski, M., Stolper, M., Sulimov, A., Susen, R., Świerblewski, J., Sydlo, C., Syresin, E., Sytchev, V., Szuba, J., Tesch, N., Thie, J., Thiebault, A., Tiedtke, K., Tischhauser, D., Tolkiehn, J., Tomin, S., Tonisch, F., Toral, F., Torbin, I., Trapp, A., Treyer, D., Trowitzsch, G., Trublet, T., Tschentscher, T., Ullrich, F., Vannoni, M., Varela, P., Varghese, G., Vashchenko, G., Vasic, M., Vazquez-Velez, C., Verguet, A., Vilcins-Czvitkovits, S., Villanueva, R., Visentin, B., Viti, M., Vogel, E., Volobuev, E., Wagner, R., Walker, N., Wamsat, T., Weddig, H., Weichert, G., Weise, H., Wenndorf, R., Werner, M., Wichmann, R., Wiebers, C., Wiencek, M., Wilksen, T., Will, I., Winkelmann, L., Winkowski, M., Wittenburg, K., Witzig, A., Wlk, P., Wohlenberg, T., Wojciechowski, M., Wolff-Fabris, F., Wrochna, G., Wrona, K., Yakopov, M., Yang, B., Yang, F., Yurkov, M., Zagorodnov, I., Zalden, P., Zavadtsev, A., Zavadtsev, D., Zhirnov, A., Zhukov, A., Ziemann, V., Zolotov, A., Zolotukhina, N., Zummack, F. & Zybin, D. (2020). *Nat. Photon.* **14**, 391–397.

[bb9] Emma, P., Akre, R., Arthur, J., Bionta, R., Bostedt, C., Bozek, J., Brachmann, A., Bucksbaum, P., Coffee, R., Decker, F.-J., Ding, Y., Dowell, D., Edstrom, S., Fisher, A., Frisch, J., Gilevich, S., Hastings, J., Hays, G., Hering, P., Huang, Z., Iverson, R., Loos, H., Messerschmidt, M., Miahnahri, A., Moeller, S., Nuhn, H.-D., Pile, G., Ratner, D., Rzepiela, J., Schultz, D., Smith, T., Stefan, P., Tompkins, H., Turner, J., Welch, J., White, W., Wu, J., Yocky, G. & Galayda, J. (2010). *Nat. Photon.* **4**, 641–647.

[bb10] Feldhaus, J., Saldin, E., Schneider, J., Schneidmiller, E. & Yurkov, M. (1997). *Opt. Commun.* **140**, 341–352.

[bb11] Freund, H. P., van der Slot, P. J. M. & Shvyd’ko, Y. (2019). *New J. Phys.* **21**, 093028.

[bb12] Geloni, G., Kocharyan, V. & Saldin, E. (2011). *J. Mod. Opt.* **58**, 1391–1403.

[bb13] Huang, Z. & Ruth, R. D. (2006). *Phys. Rev. Lett.* **96**, 144801. 10.1103/PhysRevLett.96.144801 16712082

[bb14] Inoue, I., Osaka, T., Hara, T., Tanaka, T., Inagaki, T., Fukui, T., Goto, S., Inubushi, Y., Kimura, H., Kinjo, R., Ohashi, H., Togawa, K., Tono, K., Yamaga, M., Tanaka, H., Ishikawa, T. & Yabashi, M. (2019). *Nat. Photon.* **13**, 1749–4893.

[bb15] Ishikawa, T., Aoyagi, H., Asaka, T., Asano, Y., Azumi, N., Bizen, T., Ego, H., Fukami, K., Fukui, T., Furukawa, Y., Goto, S., Hanaki, H., Hara, T., Hasegawa, T., Hatsui, T., Higashiya, A., Hirono, T., Hosoda, N., Ishii, M., Inagaki, T., Inubushi, Y., Itoga, T., Joti, Y., Kago, M., Kameshima, T., Kimura, H., Kirihara, Y., Kiyomichi, A., Kobayashi, T., Kondo, C., Kudo, T., Maesaka, H., Maréchal, X. M., Masuda, T., Matsubara, S., Matsumoto, T., Matsushita, T., Matsui, S., Nagasono, M., Nariyama, N., Ohashi, H., Ohata, T., Ohshima, T., Ono, S., Otake, Y., Saji, C., Sakurai, T., Sato, T., Sawada, K., Seike, T., Shirasawa, K., Sugimoto, T., Suzuki, S., Takahashi, S., Takebe, H., Takeshita, K., Tamasaku, K., Tanaka, H., Tanaka, R., Tanaka, T., Togashi, T., Togawa, K., Tokuhisa, A., Tomizawa, H., Tono, K., Wu, S., Yabashi, M., Yamaga, M., Yamashita, A., Yanagida, K., Zhang, C., Shintake, T., Kitamura, H. & Kumagai, N. (2012). *Nat. Photon.* **6**, 540–544.

[bb16] Kang, H.-S., Min, C.-K., Heo, H., Kim, C., Yang, H., Kim, G., Nam, I., Baek, S. Y., Choi, H.-J., Mun, G., Park, B. R., Suh, Y. J., Shin, D. C., Hu, J., Hong, J., Jung, S., Kim, S.-H., Kim, K., Na, D., Park, S. S., Park, Y. J., Han, J.-H., Jung, Y. G., Jeong, S. H., Lee, H. G., Lee, S., Lee, S., Lee, W.-W., Oh, B., Suh, H. S., Parc, Y. W., Park, S.-J., Kim, M. H., Jung, N.-S., Kim, Y.-C., Lee, M.-S., Lee, B.-H., Sung, C.-W., Mok, I.-S., Yang, J., Lee, C., Shin, H., Kim, Y., Kim, Y., Lee, J. H., Park, S., Kim, J., Park, J., Eom, I., Rah, S., Kim, S., Nam, K. H., Park, J., Park, J., Kim, S., Kwon, S., Park, S. H., Kim, S. N., Hyun, H., Kim, S. N., Kim, S., Hwang, S., Kim, M. J., Lim, C., Yu, C., Kim, B., Kang, T., Kim, K., Kim, S., Lee, H., Lee, H., Park, K., Koo, T., Kim, D. & Ko, I. S. (2017). *Nat. Photon.* **11**, 708–713.

[bb17] Kim, K.-J., Shvyd’ko, Y. & Reiche, S. (2008). *Phys. Rev. Lett.* **100**, 244802. 10.1103/PhysRevLett.100.244802 18643591

[bb18] Kim, K.-J. & Shvyd’ko, Y. V. (2009). *Phys. Rev. ST Accel. Beams* **12**, 030703.

[bb19] Kohn, V. G., Chumakov, A. I. & Rüffer, R. (2009). *J. Synchrotron Rad.* **16**, 635–641. 10.1107/S090904950902319X 19713637

[bb20] Kondratenko, A. M. & Saldin, E. L. (1980). *Part. Accel.* **10**, 207–216.

[bb21] Kujala, N., Freund, W., Liu, J., Koch, A., Falk, T., Planas, M., Dietrich, F., Laksman, J., Maltezopoulos, T., Risch, J., Dall’Antonia, F. & Grünert, J. (2020). *Rev. Sci. Instrum.* **91**, 103101. 10.1063/5.0019935 33138553

[bb22] Lengeler, B., Schroer, C., Tümmler, J., Benner, B., Richwin, M., Snigirev, A., Snigireva, I. & Drakopoulos, M. (1999). *J. Synchrotron Rad.* **6**, 1153–1167.

[bb23] Lindberg, R. R., Kim, K.-J., Shvyd’ko, Y. & Fawley, W. M. (2011). *Phys. Rev. ST Accel. Beams* **14**, 010701.

[bb24] Liu, P., Pradhan, P., Miceli, A., Walko, D. A., Shu, D., Sullivan, J., Lang, K., Rivers, M., Balcazar, M., Li, K., Margraf, R., Halavanau, A., Sakdinawat, A., Sato, T., Zhu, D. & Shvyd’ko, Y. (2024*a*). *Phys. Rev. Accel. Beams* **27**, 110701.

[bb25] Liu, P., Pradhan, P., Shi, X., Shu, D., Kauchha, K., Qiao, Z., Tamasaku, K., Osaka, T., Zhu, D., Sato, T., MacArthur, J., Huang, X., Assoufid, L., White, M., Kim, K.-J. & Shvyd’ko, Y. (2024*b*). *J. Synchrotron Rad.* **31**, 751–762. 10.1107/S1600577524003977 PMC11226151 38904936

[bb26] Liu, S., Grech, C., Guetg, M., Karabekyan, S., Kocharyan, V., Kujala, N., Lechner, C., Long, T., Mirian, N., Qin, W., Serkez, S., Tomin, S., Yan, J., Abeghyan, S., Anton, J., Blank, V., Boesenberg, U., Brinker, F., Chen, Y., Decking, W., Dong, X., Kearney, S., La Civita, D., Madsen, A., Maltezopoulos, T., Rodriguez-Fernandez, A., Saldin, E., Samoylova, L., Scholz, M., Sinn, H., Sleziona, V., Shu, D., Tanikawa, T., Terentiev, S., Trebushinin, A., Tschentscher, T., Vannoni, M., Wohlenberg, T., Yakopov, M. & Geloni, G. (2023). *Nat. Photon.* **17**, 984–991.

[bb27] Lübbert, D., Baumbach, T., Härtwig, J., Boller, E. & Pernot, E. (2000). *Nucl. Instrum. Methods Phys. Res. B* **160**, 521–527.

[bb28] Macrander, A., Erdmann, M., Kujala, N., Stoupin, S., Marathe, S., Shi, X., Wojcik, M., Nocher, D., Conley, R., Sullivan, J., Goetze, K., Maser, J. & Assoufid, L. (2016). *AIP Conf. Proc.* **1741**, 030030.

[bb30] Marcus, G., Anton, J., Assoufid, L., Decker, F.-J., Gassner, G., Goetze, K., Halavanau, A., Hastings, J., Huang, Z., Jansma, W., Kearney, S., Kim, K., Lantz, B., Lindberg, R., Liu, Y., MacArthur, J., Margraf, R., Miceli, A., Raubenheimer, T., Sakdinawat, A., Shi, X., Shu, D., Shvyd’ko, Y., Sullivan, J., Tan, T.-F., White, M. & Zhu, D. (2019). *Proceedings of the 39th Free Electron Laser Conference (FEL2019)*, Hamburg, Germany, pp. 282–287. TUD04.

[bb29] Marcus, G., Ding, Y., Duris, J., Feng, Y., Huang, Z., Krzywinski, J. T., Maxwell, T. R., Kim, K.-J., Lindberg, R., Shvyd’ko, Y. & Nguyen, D. (2017). *Proceedings of the 38th International Free Electron Laser Conference*, Santa Fe, NM, USA.

[bb31] Marcus, G., Halavanau, A., Huang, Z., Krzywinski, J., MacArthur, J., Margraf, R., Raubenheimer, T. & Zhu, D. (2020). *Phys. Rev. Lett.* **125**, 254801. 10.1103/PhysRevLett.125.254801 33416365

[bb32] Matsushita, T. & Kaminaga, U. (1980). *J. Appl. Cryst.* **13**, 465–471.

[bb33] Nam, I., Min, C.-K., Oh, B., Kim, G., Na, D., Suh, Y. J., Yang, H., Cho, M. H., Kim, C., Kim, M.-J., Shim, C. H., Ko, J. H., Heo, H., Park, J., Kim, J., Park, S., Park, G., Kim, S., Chun, S. H., Hyun, H., Lee, J. H., Kim, K. S., Eom, I., Rah, S., Shu, D., Kim, K.-J., Terentyev, S., Blank, V., Shvyd’ko, Y., Lee, S. J. & Kang, H.-S. (2021). *Nat. Photon.* **15**, 435–441.

[bb34] Pradhan, P., Wojcik, M., Huang, X., Kasman, E., Assoufid, L., Anton, J., Shu, D., Terentyev, S., Blank, V., Kim, K.-J. & Shvyd’ko, Y. (2020). *J. Synchrotron Rad.* **27**, 1553–1563. 10.1107/S1600577520012746 PMC7842206 33147180

[bb35] Qi, P. & Shvyd’ko, Y. (2022). *Phys. Rev. Accel. Beams* **25**, 050701.

[bb37] Rebuffi, L. & del Rio, M. S. (2017). *Proc. SPIE* **10388**, 103880S.

[bb36] Rebuffi, L. & Sánchez del Río, M. (2016). *J. Synchrotron Rad.* **23**, 1357–1367. 10.1107/S1600577516013837 PMC5298219 27787241

[bb39] Saldin, E., Schneidmiller, E., Shvyd’ko, Y. & Yurkov, M. (2001). *Nucl. Instrum. Methods Phys. Res. A* **475**, 357–362.

[bb38] Sanchez del Rio, M., Canestrari, N., Jiang, F. & Cerrina, F. (2011). *J. Synchrotron Rad.* **18**, 708–716. 10.1107/S0909049511026306 PMC3267628 21862849

[bb40] Shvyd’ko, Y. (2004). *X-ray Optics – High-Energy-Resolution Applications*, Vol. 98 of *Optical Sciences.* Berlin, Heidelberg: Springer.

[bb43] Shvyd’ko, Y. (2015). *Phys. Rev. A* **91**, 053817.

[bb44] Shvyd’ko, Y., Röhlsberger, R., Kocharovskaya, O., Evers, J., Geloni, G. A., Liu, P., Shu, D., Miceli, A., Stone, B., Hippler, W., Marx-Glowna, B., Uschmann, I., Loetzsch, R., Leupold, O., Wille, H.-C., Sergeev, I., Gerharz, M., Zhang, X., Grech, C., Guetg, M., Kocharyan, V., Kujala, N., Liu, S., Qin, W., Zozulya, A., Hallmann, J., Boesenberg, U., Jo, W., Möller, J., Rodriguez-Fernandez, A., Youssef, M., Madsen, A. & Kolodziej, T. (2023). *Nature* **622**, 471–475. 10.1038/s41586-023-06491-w PMC10584683 37758953

[bb42] Shvyd’ko, Y., Stoupin, S., Mundboth, K. & Kim, J. (2013). *Phys. Rev. A* **87**, 043835.

[bb41] Shvyd’ko, Y. V., Lerche, M., Kuetgens, U., Rüter, H. D., Alatas, A. & Zhao, J. (2006). *Phys. Rev. Lett.* **97**, 235502. 10.1103/PhysRevLett.97.235502 17280212

[bb45] Siegman, A. E. (1986). *Lasers.* Sausalito: University Science Books.

[bb46] Snigirev, A., Kohn, V., Snigireva, I. & Lengeler, B. (1996). *Nature* **384**, 49–51.

[bb47] Stoupin, S., Shvyd’ko, Y., Trakhtenberg, E., Liu, Z., Lang, K., Huang, X., Wieczorek, M., Kasman, E., Hammonds, J., Macrander, A. & Assoufid, L. (2016). *AIP Conf. Proc.* **1741**, 050020.

[bb48] Terentyev, S., Blank, V., Kolodziej, T. & Shvyd’ko, Y. (2016). *Rev. Sci. Instrum.* **87**, 125117. 10.1063/1.4973326 28040980

[bb49] Yabashi, M., Hastings, J. B., Zolotorev, M. S., Mimura, H., Yumoto, H., Matsuyama, S., Yamauchi, K. & Ishikawa, T. (2006). *Phys. Rev. Lett.* **97**, 084802. 10.1103/PhysRevLett.97.084802 17026309

[bb50] Zhu, D., Cammarata, M., Feldkamp, J. M., Fritz, D. M., Hastings, J. B., Lee, S., Lemke, H. T., Robert, A., Turner, J. L. & Feng, Y. (2012). *Appl. Phys. Lett.* **101**, 034103.

